# Influence of Gd doping on Cu_2_Sn_1-x_Gd_x_S_3_ thin film solar cell

**DOI:** 10.1016/j.isci.2025.112597

**Published:** 2025-05-06

**Authors:** Şilan Baturay, Serap Yiğit Gezgin, M. Zafer Köylü, Mohamed A. Basyooni-M. Kabatas, Hamdi Şükür Kiliç

**Affiliations:** 1 Department of Physics, Faculty of Science, Dicle University, Diyarbakir 21280, Turkey; 2 Department of Physics, Faculty of Science, University of Selcuk, Selcuklu, Konya 42031, Turkey; 3 Dynamics of Micro and Nano Systems Group, Department of Precision and Microsystems Engineering, Delft University of Technology, Mekelweg 2, Delft 2628 CD, the Netherlands; 4 Department of Nanotechnology and Advanced Materials, Graduate School of Applied and Natural Science, Selçuk University, Konya 42030, Turkey; 5 Solar Research Laboratory, Solar and Space Research Department, National Research Institute of Astronomy and Geophysics, Cairo 11421, Egypt; 6 Department of Metallurgy and Materials Engineering, Faculty of Engineering, University of Dokuz Eylül, İzmir, Turkey

**Keywords:** Chemistry, Energy engineering, Materials science

## Abstract

The effect of the Gd/Sn composition ratio of Cu_2_Sn_1-x_Gd_x_S_3_ is examined. The films are fabricated on glass substrates in a sulfur atmosphere via the spin coating. The influence of the Gd/Sn composition ratio on the structural, morphological, optical, and electrical properties of the films is investigated using X-ray diffraction, FESEM, UV-Vis, and Hall effect. XRD patterns for the films revealed that all films have a monoclinic polycrystalline. The morphological and optical properties of the films show the formation of spherical grains and polygonal structures with an energy band in the range of 2.10–1.50 eV. The electrical properties of the films are changed by increasing the Gd/Sn composition ratio in the film. Furthermore, the Cu_2_Sn_1-x_Gd_x_S_3_/CdS heterojunction solar cell was modeled by SCAPS-1D. The optimized conditions yielded exceptional photovoltaic parameters, achieving an open-circuit voltage of 0.7885 V, short circuit current density of 41.09 mA/cm^2^, fill factor of 85.30%, and an efficiency of 27.3%.

## Highlights

• Gd-doped Cu₂SnS₃ films made by the one-step spin coating method
• Gd/Sn ratio affects structural, optical, and electrical properties
• Films show grains with band gaps ranging from 2.10 to 1.50 eV
• SCAPS-1D modeled Cu/p-Gd:CTS/CdS/ZnO/ITO solar cell structure

## Graphical abstract

**Figure:**
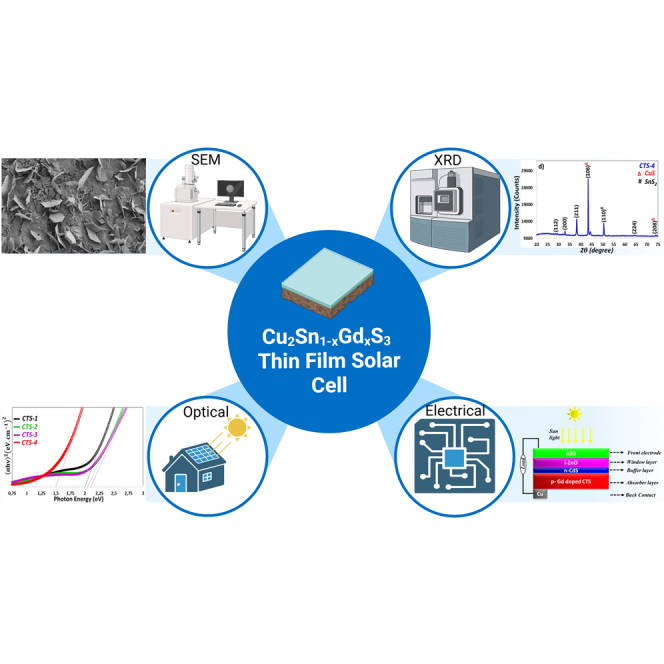

## Introduction

The global demand for renewable energy has spurred significant interest in producing photovoltaic cells to convert solar energy into electricity. Lowering manufacturing costs and the weight of these modules is crucial to expediting installation. Regarding this matter, the leading polycrystalline thin-film solar cells, such as polycrystalline Cu(In, Ga)Se_2_ (CIGS) and CdTe, have exhibited impressive photovoltaic conversion efficiencies of 22.0% Cu(In, Ga)Se_2_ for small areas and 22.1% and have captured around 5% of the current world photovoltaic (PV) market, respectively.^1^^,^^2^ Nevertheless, the above-mentioned absorb layers include scarce elements (In, Ga, Se, and Te) and toxic elements (Te, Cd, and Se) that limit their future applications. Alternatively, the replacement of “Ga,” “Se,” and “In” by environmentally friendly, greatly plentiful, and low-cost elements such as “Zn,” “S,” and “Sn” results in the improvement of a new and potentially useful absorber material, Cu_2_ZnSnS_4_.^3^ Even though Cu_2_ZnSnS_4_ solar cells exhibited a record conversion efficiency of 11% with a high open-circuit voltage ($V_{OC}$),^4^ the fabrication of single-phase Cu_2_ZnSnS_4_ is challenging because of its complicated structure and phase diagram with a narrow window for a single-phase structure.

The critical challenges are detecting and suppressing secondary phases, including Cu–S, Sn–S, Zn–S, and Cu–Sn–S in obtained Cu_2_ZnSnS_4_ films related to processing conditions. Several attempts have been made to eliminate these secondary phases through post-fabrication chemical etching procedures.^5^ The formation of single-phase CZTS was not obtained because chemical etching only removes secondary phases on the surface, leaving those within the interior intact. Additionally, controlling the “Zn” composition during the fabrication of CZTS films is particularly challenging due to the higher vapor pressure of Zn compared to Cu, Sn, and S. Moreover, Zn-related defects are predominantly deep-level "killer defects," which significantly contribute to the poor performance of CZTS solar cells.^6^^,^^7^ Secondary phases can dramatically impact the photovoltaic device’s performance. If these phases consist of wide band gap materials, such as ZnS, they reduce the volume of the absorber layer with the ideal energy band gap for single-junction photovoltaic cells. This reduction consequently leads to a decrease in short-circuit density ($J_{SC}$). Additionally, ZnS, a secondary phase that is an *n*-type semiconductor, can form anywhere within the CZTS, hindering carrier transport and increasing recombination. Growing ternary Cu–Sn–S thin films, which do not contain Zn, is more straightforward in solving these difficulties. The control of structure and defects in Cu–Sn–S samples is also easier than in CZTS, as Cu–Sn–S samples consist of only three elements.

In 1987, Kuku et al. reported the first CTS-based solar cell, achieving a conversion efficiency of 0.11% using a direct evaporation technique.^8^ CTS films can exist in various crystallite structures, including cubic, tetragonal, triclinic, and monoclinic.^9^^,^^10^^,^^11^^,^^12^ The tetragonal and cubic phases achieved the highest power conversion efficiencies (PCEs of 8% and 10%, respectively, with 2500 nm optimum absorber layer thickness and charge carrier concentrations of 10^18^/cm^3^. In contrast, the orthorhombic phase required a slightly thicker absorber layer of approximately 3000 nm, yielding a PCE of 12%.^13^ The different structures of ternary CTS films have been grown recently using a variety of fabrication techniques, including physical vapor deposition,^14^ a single-step electrochemical deposition process,^15^ chemical bath deposition,^10^ ultrasonic spray pyrolysis,^16^ sputtering,^17^ and spin coating.^11^ Unlike other fabrication methods, spin-coating is particularly efficient because of its low cost, high fabrication rate, and different fabrication processes, including film thickness and substrate temperature. Although the physical and chemical behaviors of Cu_2_SnS_3_ films have been investigated, the electrical properties and Solar Cell Capacitance Simulator (SCAPS-1D) calculations of Cu_2_Sn_1-x_Gd_x_S_3_ films prepared via the spin-coating technique have not yet been comprehensively studied. Therefore, experimental and theoretical analyses are essential to better understand gadolinium’s influence on the physical behavior of CTS film. There is a lack of sufficient knowledge regarding these effects on crystallite parameters, energy band gap, and SCAPS-1D simulations of Cu_2_Sn_1-x_Gd_x_S_3_ (where x = 0, 0.25, 0.50, and 0.75) samples were produced through spin-coating. The simulation tool employed in this study, SCAPS-1D, is a Windows-based application developed at the University of Gent using LabWindows/CVI from National Instruments. SCAPS-1D software was initially designed to model CdTe, CuInSe_2_, and CIGS thin film solar cells in one dimension.^18^ Its most helpful feature is its ability to compute AC and DC electrical characteristics under various light intensities and temperature conditions.^19^^,^^20^ The SCAPS program can implement some special features, such as the simulation of the impurity levels, interfaces of photovoltaic devices.^21^ To validate our model and explore the effects of different Gd doping on solar cell performance, we conducted calculations for both the Cu_2_SnS_3_ sample and Cu_2_Sn_1-x_Gd_x_S_3_ (where x = 0.25, 0.50, and 0.75) samples. Key parameters analyzed included the $V_{OC}$, $J_{SC}$, and FF.

However, there is limited literature on tuning the optical energy gap and enhancing the crystalline quality of Cu_2_SnS_3_ thin films. Recently, Umehara et al. synthesized cubic Cu_2_Sn_0.85_Ge_0.17_S_3_ thin film, followed by a sulfurization process in the furnace. The partial substitution of Ge for Sn in CTS successfully tuned the energy gap between 0.93 eV and 1.02 eV and significantly enhanced the grain size of the thin film.^13^ Doping Cu_2_SnS_3_ with Ge is a promising approach to improve its efficiency and stability in photovoltaic devices. Ge modifies the electronic structure of the material and charge carrier concentration, thereby influencing its overall semiconductor properties.^22^ This impressive study demonstrates a practical approach to enhancing the efficiency of CTS solar cells by selecting appropriate dopant cations to tune the energy band and improve the crystalline quality of CTS thin films.

The Gd^3+^ ion has a larger ionic radius than Cu^2+^ and Sn^4+^ ions. Consequently, doping CTS with Gd is expected to facilitate forming a solid solution incorporating Gd^3+^, potentially leading to the development of Cu-Sn-S-Gd alloy or Cu_2_GdSnS_3_ thin films. Since Cu_2_SnS_3_ has optical energy band value of around 0.95 eV for cubic structure, while Cd doped Cu_2_SnS_3_ thin film has optical energy band value of around 1.37 eV and Ge doped Cu_2_SnS_3_ thin film has optical energy band value of 1.23 eV, it is likely to gain the energy band value in the different range by doping.^23^^,^^24^ Doping Cu_2_SnS_3_ with Ge/Sn co-doping is a possible technique to enhance its stability and efficiency in performance in solar cell devices. Ge is known for modifying the physical properties of semiconductors by altering their charge carrier concentration and electronic structure.^22^

Additionally, in solution-processed thin films, Ge doping may impact the growth and morphology of the film, as well as its optoelectronic properties. The value of power conversion efficiency was improved by the adding of Na, Sb, and Na/Sb co-doping to the CTS absorber layer, from 0.32% for non-doped CTS to 0.76%, 1.54%, and 1.89 for co-doping, Na, and Sb doped CTS, respectively.^25^ In this context, examining the effect of different doping on the physical properties of sol-gel processed Cu_2_SnS_3_ thin films is crucial for understanding how to improve the working performance of Cu_2_SnS_3_-based thin-film solar cells. The present study investigates the effects of Gd concentration on the physical properties and solar cell parameters of Cu-Sn-S-Gd thin films for the fabrication of high-quality Gd^3+^ doped Cu_2_SnS_3_ films via spin coating followed by sulfurization in a quartz furnace. The work presented a powerful method to tune the energy gap of Cu_2_SnS_3_ thin films and a potential approach to improve the efficiency of Cu_2_GdSnS_3_ and Cu_2_SnS_3_ thin film solar cells.

Researchers have used chemical and physical deposition techniques for growing CTS samples related to different deposition parameters such as substrate temperature, film thickness, and annealing time. CTS films with low Cu concentration indicated a suitable energy band gap value in solar cells with high crystallinity. All obtained CTS films displayed *p*-type electrical conductivity, decreasing resistivity as the Cu concentration increased in several CTS phases.^26^ Solar power conversion efficiencies of 0.51% have been gained experimentally for monoclinic CTS solar cells after sulfurization for 10 min.^27^ The structure of Mo/CTS/CdS/i-ZnO/AZO/Al thin film solar cell also indicates an efficiency of 1.35% with the short circuit current density ($J_{SC}$) of 28.3 mA/cm^2^, open circuit voltage ($V_{OC}$) of 147.5 mV, and fill factor (FF) of 32%.^28^ In addition, theoretical research suggests that CTS-based photovoltaic devices can achieve an efficiency of 17–20%.^29^^,^^30^ Monnaf et al. indicated that the structure of Ni/V_2_O_5_/Cu_2_SnSe_3_/In_2_S_3_/ITO/Al novel heterostructure thin-film solar cell using the SCAPS-1D program suggests the efficiency of up to 32.34% can be gained by the hole transport layer and electron transport layer combination of V_2_O_5_ and In_2_S_3_, respectively.^31^ Consequently, the full potential of CTS has not yet been explored for use in thin-film solar cells.

Studies on solar cells produced based on *p*-type CTS active layer are limited. Therefore, this study employs CTS samples as absorber layers to reveal their hidden prospects. Determining the performance of the Cu/*p*-Cu_2_Sn_1-x_Gd_x_S_3_/*n*-CdS/i-ZnO/ITO thin film solar cell theoretically before solar cell production always leads to experimental studies. This study investigated the morphology, crystal, optical, and electrical properties of the effect of Gd element on CTS material. The optimal *p-*Gd doped CTS material was determined, and the solar cell based on this material was modeled. Depending on the parameters such as interface defect density, acceptor defect density, electron affinity, radiative recombination coefficient, Auger electron capture coefficient, series, and shunt resistance, the highest photovoltaic parameters were determined for the best conditions, and the lower ones for the less favourable conditions. The modeled Cu/*p*-Cu_2_Sn_1-x_Gd_x_S_3_/*n*-CdS/i-ZnO/ITO thin film solar cells have been computationally analyzed using SCAPS-1D software to explore their potential in advancing photovoltaic technology.

## Results and discussion

### Structural properties

**Figure 1:**
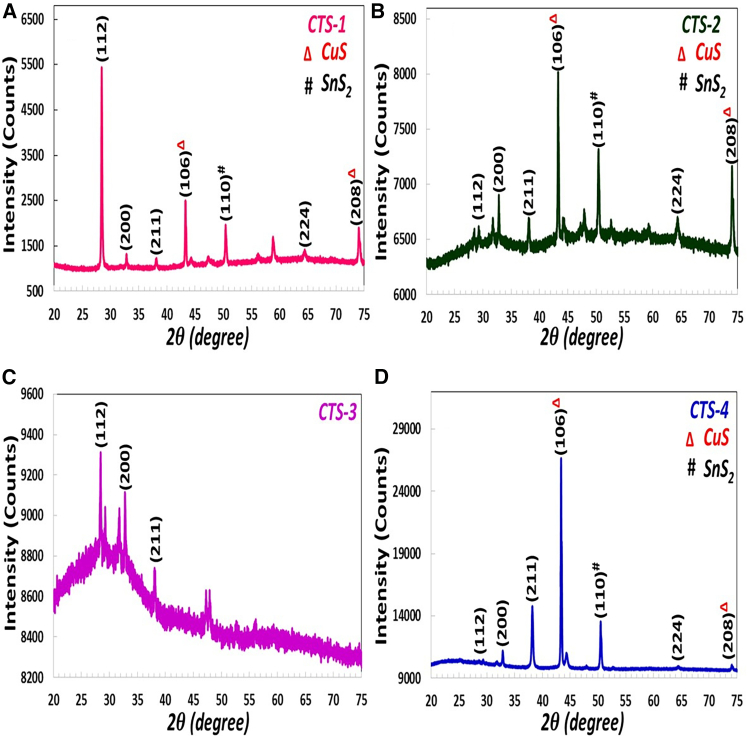
XRD Analysis X-ray diffraction pattern of (A) CTS-1, (B) CTS-2, (C) CTS-3, and (D) CTS-4 thin films.

Figure 1 shows the X-ray diffraction (XRD) patterns of the Cu_2_Sn_1-x_Gd_x_S_3_ (where x = 0, 0.25, 0.50, and 0.75) annealed at 500°C. In addition, the Cu_2_Sn_1-x_Gd_x_S_3_ fabricated at annealing temperatures of 500°C are denoted as CTS-1, CTS-2, CTS-3, and CTS-4, respectively. CTS-1, CTS-2, and CTS-3 films exhibited diffraction peaks at around 2θ = 28.44°, 32.64°, 38.28°, 43.20°, 50.29°, 58.92°, and 74.19°, which correspond to the (112), (200), (211), (106), (110), (224), and (208) diffraction planes, respectively. The intensity of the (106) peak belonging to the single covellite phase, a hexagonal crystal structure, significantly changed owing to a change in the Sn ratio and disappeared in the CTS-3 thin film due to the low melting point of tin during the annealing process. Furthermore, it can be seen from Figure 1A that the CTS-1 thin film indicated the typical peak of Cu_2_SnS_3_ emerging at 2θ = 28.44° and corresponding to the (112) diffraction plane. It gradually decreased and vanished. This is because the change of Sn becomes more severe as the increase in Cu/Sn atomic ratio (given in Figures 3A–3D) with an annealing effect prevents the formation of phases containing Sn. The formation of this phase can be explained by the presence of tin, which undergoes a reaction that gives rise to a copper and sulfur-rich environment during the annealing process of the obtained thin films. This occurs due to the low melting point of tin, leading to the formation of secondary phases, including CuS and SnS_2_. The presence of secondary phases in the annealed thin films resulted in a significant change in both the peak positions and peak intensities. However, when the Gd doping increased to 0.50 (Gd/Sn = 1), the distinctive peak of CuS at around 2θ = 43.20° vanished, and the Gd/Sn doping concentration was more significant than 1, and the CuS peak appeared. It can be concluded that adjusting the tin concentration alters the elemental composition of the films, consequently affecting the crystallinity of the CTS thin films. The crystallite size (D) of CTS thin films is carefully calculated for two prominent (112) and (106) peaks by Debye-Scherrer’s formula^32^;

(Equation 1) $$D=\frac{0.94\lambda }{\beta \;\cos\;\theta }$$

Where λ is the wavelength of the X-ray diffractometer, θ and β are the Bragg diffraction angle and full-width half maximum (FWHM) value of XRD peaks, respectively. The value of crystallite size is found to be 53.37 and 51.59 nm for (112) orientation for CTS-1 and CTS-3 thin film, respectively, and 43.30, 50.30, and 45.87 nm for (106) orientation for CTS-1, CTS-2, and CTS-4 thin film, respectively. The change in the crystallite size value might be due to increased crystallite defects and lattice mismatching between the host and dopant ions. Based on FE-SEM images, particle size calculations confirm the changing behavior of crystallite sizes. The peak narrowing indicates the formation of nanocrystals, and the central peak of any spectrum supports that the obtained CTS films have a crystalline nature with different crystal orientations. Observing the magnified (112) and (110) diffraction peaks confirmed that the position of each peak is slightly shifted toward the upper region. This figure shows that for the CTS-1 film, the predominant phase is Cu2SnS3, which has a cubic structure.^33^ CTS-3 sample showed significant peaks at diffraction peaks at around 2θ = 28.44°, 32.64°, and 38.28°, which correspond to the (112), (200), and (211) diffraction planes, respectively. The peaks in cubic Cu_2_SnS_3_ had decreased their peak intensity. The intensity of the (106) peak belonging to the single covellite phase disappeared due to the preference for tin-containing interactions, which are influenced by the interaction between tin and other materials. From Cu_2_SnS_3_ XRD peaks, additional peaks corresponding to the secondary phase, including copper sulfate (CuS) (indicated by Δ) and tin sulfide (SnS_2_) (indicated by #), were observed in the diffraction pattern. In the CTS film, the CuS secondary phase displays the single covellite phase with a hexagonal crystal structure,^34^ and the CTS-1, CTS-2, and CTS-3 samples are a multi-phase containing CuS, SnS_2_, and Cu_2_SnS_3_ with a cubic structure.

**Figure 2:**
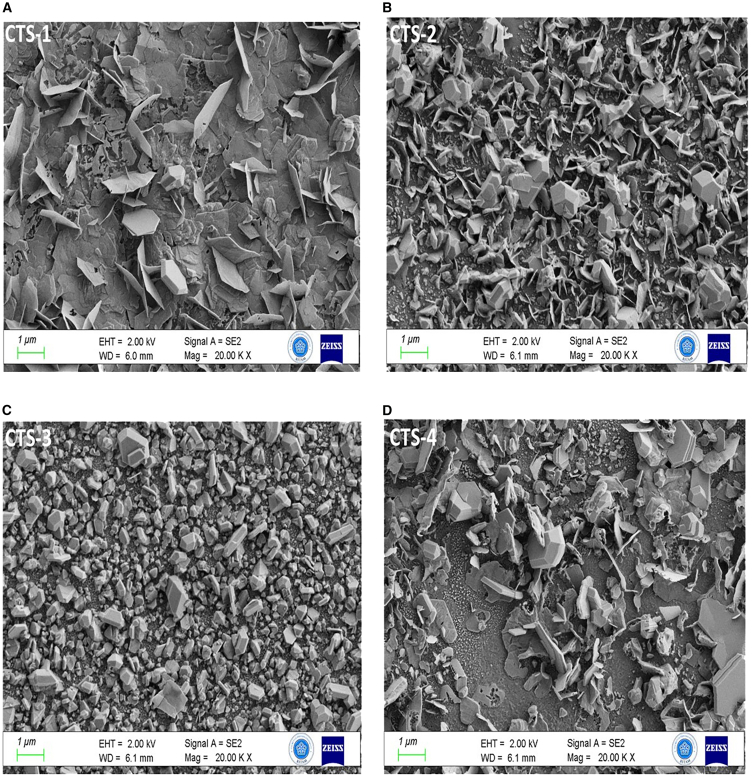
Surface Morphology FE-SEM images of (A) CTS-1, (B) CTS-2, (C) CTS-3, and (D) CTS-4 thin films.

FE-SEM studies indicate essential information on the morphology of the fabricated thin films. It gives crucial insights into particle shape, grain size, and topology on the surfaces of fabricated thin films. The use of low concentration during Cu_2_Sn_1-x_Gd_x_S_3_ (where x = 0, 0.25, 0.50, and 0.75) preparation has been associated with the generation of smaller grain sizes.^35^^,^^36^ As the Gd/Sn concentration changes, a notable change in the surface topology of the obtained CTS films becomes clear, as given in Figure 2. Figure 2 illustrates the obtained samples produced on glass using the spin coating technique, sulfurized at 500°C sulfur and in an Argon atmosphere. Characteristically, the composition of the obtained samples consists of spherical grains and polygonal structures. However, with increasing Gd concentration in solution, a pronounced transformation happens, causing the structure of larger clusters within the samples and an essential shift in surface topology. These samples exhibit densely packed grain morphology resembling nanosheet or flake morphology, indicating that annealing and Gd concentration in the solution induce nucleation and introduce surface quality modifications.^37^ The broken and unbroken nanosheet structure can be observed clearly, as shown in Figure 2, from the samples sulfurized at 500°C in a sulfurous atmosphere. Images obtained by scanning electron microscopy reveal a panoramic view of the samples, composed of numerous polygonal and compact nanosheet structures. In this context, the agglomeration of Cu_2_Sn_1-x_Gd_x_S_3_ nanoparticles results in predominant and uniform grain growth. When Gd ratios in a solution are higher, crystalline grains are more easily amalgamated, resulting in bigger particles and better uniformity. Consequently, the increase in Gd ratio reduces charge concentration and energy band gap, leading to enhanced device performance.

**Figure 3:**
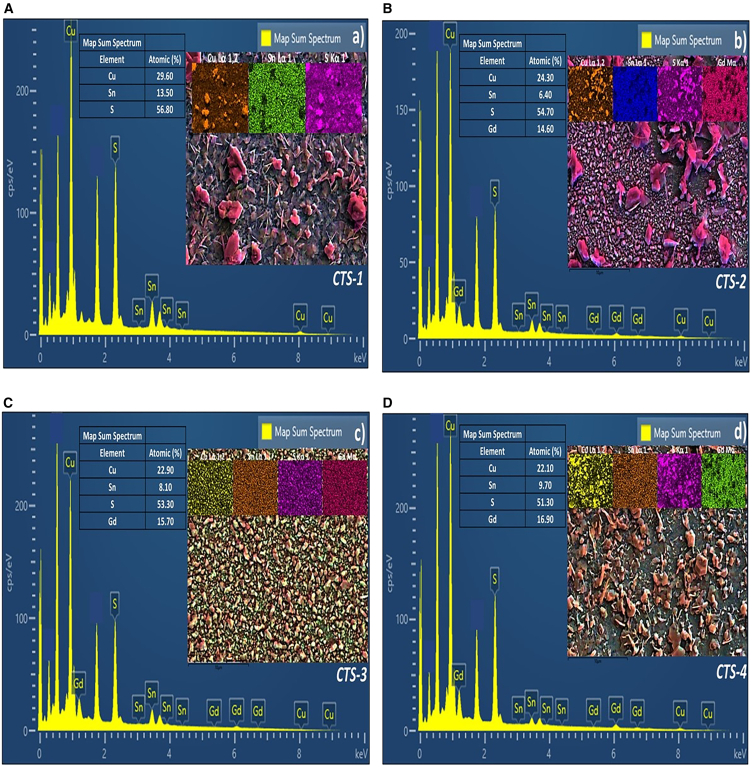
Elemental Mapping Elemental mapping of (A) CTS-1, (B) CTS-2, (C) CTS-3, and (D) CTS-4 thin films.

The atomic percentage of the CTS films is presented in Figures 3A–3D. As shown in Figure 3, all obtained thin films revealed Sn-poor composition, as targeted. As shown in Figure 3, CTS-1, CTS-2, and CTS-3 films indicated a changing Cu/Sn atomic ratio between 2.19 and 3.80. The difference in the thin films' Cu/Sn atomic ratio may be attributed to Sn-loss resulting from the increasing Gd/Sn doping ratio. The S/(Cu+Sn+Gd) atomic ratio was 1.32, 1.21, 1.41, and 1.05 for CTS-1, CTS-2, CTS-3, and CTS-4 films, respectively. This ratio indicated more than the desired for stoichiometric Cu_2_SnS_3_ thin film. It is believed that the excessive amount of sulfur is due to the gradual cooling of the quartz furnace over a long period after the high-temperature reaction of CTS samples and the enclosed nature of the environment.

### Optical properties

**Figure 4:**
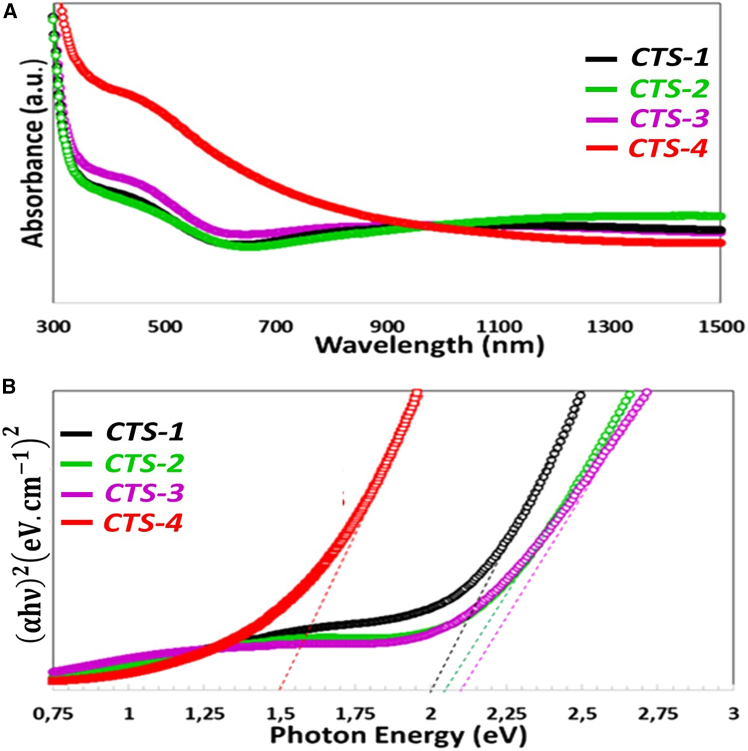
Optical Absorption (A) Absorbance and (B) Tauc Plot of CTS thin films.

UV-vis data collected between 300 and 1100 nm were used to examine the absorbance wavelength of all thin films. The absorbance-wavelength graphs of all the samples that were vacuum-annealed in a quartz furnace for 45 min at 500°C are displayed in Figure 4A. It is apparent from the figure that absorbance is found to rise with the increasing Gd doping concentration, mostly because of the reduction of the secondary phases. And it could be higher than that of the IR region. The absorbance rises with the changing Gd/Sn ratio in the solution, mainly because of the change in the secondary phases of CuS and SnS_2_. It should be stated that the presence of secondary phases changes the optical behavior. Gd-incorporated thin films have a lower decreasing absorption tendency than the non-doped CTS thin films in the visible region.

In contrast, Gd molar concentration increases, non-doped particle size increases (Figure 2), and film transparency decreases. Thus, the absorption density of these samples increases, as seen from absorbance-wavelength spectra in the visible region. It displays the deviation of the energy band gap of Gd-added samples from pure CTS samples.

The well-known Taug relationship, which is represented as Equation 2, can be used to determine the energy band gap ($E_{g}$) of the obtained thin film

(Equation 2) $$\alpha h\nu =A{\left(h\nu -E_{g}\right)}^{n}$$

In this case, hυ represents the incident photon energy, and A is a constant. The absorption coefficient in cm^−1^ is indicated by the constant α. The exponent n, which is equivalent to ½ for allowed direct transitions and 2 for allowed indirect transitions, is dependent upon the type of transition. It is commonly known that thin films based on CTS have a direct energy band gap, which can be computed by applying the n = ½ value.^38^ The energy band gap of the obtained thin film is generated by extrapolating the linear line of ${\left(\alpha h\nu \right)}^{2\;}$ vs. hυ in Tauc plot as indicated in Figure 4B. The energy band gap for the obtained samples changed between 2.10 eV and 1.50 eV, which is related to the Gd ratio in the Cu_2_SnS_3_ solution. Due to the transition to nano-grain size, the high energy band gap values in CTS films were seen from the shift toward the shorter wavelengths region, which led to enhancing energy band gap. As the FE-SEM images display the growth of nanosized particles, the quantum confinement effect on the energy band gap was ruled out.^38^ Therefore, these differences in the energy band values are caused by stoichiometry variations and secondary phases.^39^ Obtained CTS thin films annealed at high temperatures at a quartz furnace increase Sn’s evaporation because of its low melting point, and the Sn deficient condition results in the formation of another CTS phase. Compared to the accepted value of 1.3 eV for CTS film, the values in this study are 2.0, 2.05, 2.10 eV, and 1.50 eV, which can be attributed to a mixture of SnS_2_ and CuS secondary phases in the obtained samples (as seen in XRD). Generally, the secondary phase enhances the energy band gap values.^40^

The great values of energy band gap indicate interesting physical behavior, such as increasing the semiconductor energy gap because of electron confinement, which is characterized by the nanoscale systems,^41^ or variations in phase and stoichiometry rather than the quantum size effect.^42^ The energy band gap of Cu_2_SnS_3_ thin films was reported between 2.00 and 2.23 eV in many studies, and the findings of this investigation are compatible with these studies. The low value of the energy band in CTS-4, as compared to pure CTS, is owing to the tail-like effect. In addition, a further increase in Gd/Sn doping concentration resulted in a reduced energy band gap. This can be attributed to incorporating Gd^3+^ ions in the CTS lattice, forming donor states within the energy band. As the concentration of Gd^3+^ ions in the CTS lattice increases, these donor states evolve into degenerate levels, causing an expansion of the conduction band in CTS, thereby reducing the energy band gap. The decrease in the energy band gap could be due to the increase in donor density when Gd is added to the CTS nanostructures. The reduction in the energy band gap may cause the formation of a band-tailing effect.

### Electrical properties

The electrical characterization of the CTS samples is measured via the Hall effect system along with the van der Pauw technique. Table 1 indicates the measured electrical properties of the Cu_2_Sn_1-x_Gd_x_S_3_ (where x = 0, 0.25, 0.50, and 0.75) samples produced on glass substrates by the spin coating. So far, the electrical characterizations of non-doped CTS are given for thin films using various fabrication techniques by different research groups.^23^^,^^24^ However, this study was carried out at 300 K with Gd-incorporated thin films, which will be advantageous in understanding the Gd effect in the CTS film. The copper and sulfur concentrations stayed constant for all the thin films, and the tin and gadolinium concentrations changed throughout the study. The hall coefficient of the thin films is found to be positive, which indicates *p*-type conductivity. CTS-1 sample indicates a free hole concentration of the order of 10^20^ cm^−3^. After Gd was incorporated into thin films, it decreased to 10^18^ cm^−3^ because the gadolinium in the CTS solution caused the decrease in the hole concentration of the pure Cu_2_SnS_3_ film owing to its high melting point. However, the increase in Gd concentration in the solution did not affect the charge concentration.

**Table 1:** Electrical parameters of CTS-1, CTS-2, CTS-3, and CTS-4 films

Sample	$E_{g}$ (eV)	Carrier concentration (cm^−3^)	Mobility (cm^2^/VS)	Resistivity (Ω-cm)
CTS-1	2.00	2.88 × 10^20^	1.19	18.15 × 10^−3^
CTS-2	2.05	2.76 × 10^18^	35.44	12.78 × 10^−2^
CTS-3	2.10	6.34 × 10^18^	18.48	10.66 × 10^−2^
CTS-4	1.50	4.12 × 10^18^	59.83	50.60 × 10^−2^

Furthermore, changes in the chemical potential can significantly impact the charge carrier concentration, which can be managed by doping or other techniques.^42^ The maximum carrier concentration and the minimum resistivity value are observed for CTS-1 thin film, which can be attributed to the conductive Cu metallic-like phase that likely formed because of its Cu and Sn-rich composition. All the other films (CTS-2, CTS-3, and CTS-4), which are determined to be Sn-poor, display significantly reduced carrier concentration values and higher resistivity values compared to the CTS-1 thin film. Nonetheless, these values of CTS-2, CTS-3, and CTS-4 thin film in this study are in the region of 10^18^ cm^−3^, indicating semiconducting property. Values measured for CTS-1, CTS-2, and CTS-3 thin films are significantly lower than CTS-1 (10^20^ cm^−3^), and Gd doping concentration in CTS makes the thin film suitable for photovoltaic applications. The co-existence of secondary CuS and SnS_2_ phases, which can be characterized as impurity phases in the CTS thin films, could contribute to the low carrier concentration.

Here, it was seen that with the addition of the gadolinium, the film indicates elemental tin loss and gives rise to a change in the electrical parameters of the Cu_2_SnS_3_ sample. The resistivity of the obtained films changed between 18.15 × 10^−3^ Ω-cm and 50.60 × 10^−2^ Ω-cm. This change may be attributed to stoichiometric changes. The carrier concentration of the films decreased considerably with Gd; the increase in resistivity is attributed to a decrease in charge concentration attributed to the exchange of Gd^3+^ ions with Cu^2+^ ions in the CTS lattice, creating one free electron. Srinivasa et al.^43^ indicated that the charge concentration, mobility, and resistivity of CTS samples fabricated at 250°C are 2.81 × 10^21^ cm^−3^, 1.70 cm^2^ V^−1^S^−1^, and 1.31 × 10^−3^ Ω-cm, respectively.

The mobility is increased from 1.19 to 59.83 cm^2^/VS with increased Gd. The increase in mobility value (electron transport) with rising Gd is attributed to the structure of non-compact topologies such as voids, pinholes, and grain boundaries (Figure 2) and the effects of the energy band gap, leading to a change in electrical conductivity.^42^ Therefore, the observed decrease in resistivity and increase in mobility in this study are strongly correlated with changes in crystallinity and grain size in CTS films.^44^ The increased crystallinity with increasing Gd doping in pure CTS film (Figure 1), owing to the effect of Ostwald ripening, causes a decrease in the grain boundaries and a subsequent increase in the value of mobility.^45^ Thus, it is concluded that the electrical properties of the CTS films depend on the rise in gadolinium in the CTS. The 10^18^ cm^−3^ and 59.83 cm^2^/VS values are consistent with those calculated for the CuS thin film.^46^ These results can be attributed to the increase in the (106) oriented CuS phase with the rise in the Gd/Sn ratio, as also observed in the XRD analysis. The investigated values of this study agree with previous research^47^ and are more appropriate for thin-film solar cells.

### Solar cell capacitance simulator simulation

A simulation program is needed to verify the efficiency of experimentally produced solar cells or to model a possible solar cell by combining the produced semiconductor thin films. SCAPS-1D, AMPS-1D, SILVACO, and ATLAS simulation programs are used to calculate and verify the potential efficiency of solar cells. SCAPS-1D calculates the efficiency of solar cells consisting of multiple layers outside of conventional methods.

**Figure 5:**
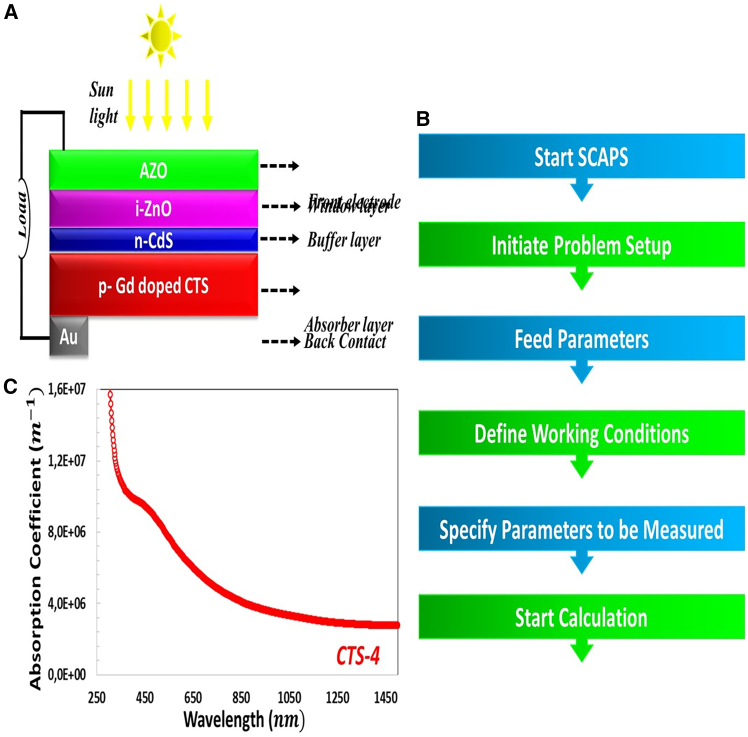
Device Design Overview (A) The basic structure of the CTS-4 solar cell, (B) the work process of the SCAPS-1D simulation program, and (C) the absorption coefficient of the CTS-4 thin film.

In this study, Back Contact/*p*-Cu_2_Sn_1-x_Gd_x_S_3_/*n*-CdS/i-ZnO/ITO, the solar cell was modeled based on the CTS-4 absorber layer with the SCAPS-1D program. SCAPS-1D software solves the fundamental semiconductor equations that govern charge transport within the material. SCAPS-1D program offers grading of all input parameters such as recombination type, charge type, defect levels (bulk and interfacial), defect level distribution containing Gaussian, single level, uniform, or combinations, contact behaviors (flat band or work function), optical properties (direct excitation of light). The performances of thin film solar cells, including fill factor, open-circuit voltage, and short-circuit current density, are significantly impacted by the back contact.^48^ Figures 5A and 5B show the thin-film solar cell basic structure and working process of the SCAPS-1D program. The physical parameters of the layers forming CTS-4 solar cells are given in Table 2. The file of the absorption coefficient of the CTS-4 thin film was input into the software program.

**Table 2:** The physical parameters of the layers of the modeled CTS-4 solar cell

Layers for solar cells	AZO [17]	i-ZnO [18]	CdS (Rafee Mahbub et al., 2016)	CTS-4
Energy Gap (eV)	3.3	3.3	2.4	1.5 (exp.)
Affinity of electron (eV)	4.6	4.6	4.2	3.7
The permittivity of dielectric (relative)	9	9	10	9.5
States CB effective density (cm-3)	2.20 × 10^18^	2.20 × 10^18^	2.20 × 10^18^	2.20 × 10^18^
States VB effective density (cm-3)	1.80 × 10^19^	1.80 × 10^19^	1.80 × 10^19^	1.80 × 10^19^
Thermal velocity of electron/Hole (cm/s)	1.00 × 10^7^	1.00 × 10^7^	1.00 × 10^7^	1.00 × 10^7^
mobility of electron/Hole (cm2/Vs)	100/25	100/25	100/25	100/59.82(exp.)
Density of shallow donor (cm-3)	1.00 × 10^20^	1.00 × 10^5^	1.00 × 10^18^	0
Density of shallow acceptor (cm-3)	0	0	0	4.12 × 10^18^ (exp.)
Thickness of film (nm)	100	100	50	500 (exp.)

The program performs the calculation of photovoltaic parameters using the following equations (Equation 3):

(Equation 3) $$\frac{d\gamma }{dx}=-\frac{d^{2}\Psi }{dx^{2}}=\frac{q}{\varepsilon }\left[p\left(x\right)-n\left(x\right)+{N_{D}}^{+}\left(x\right)-{N_{A}}^{-}+p_{t}\left(x\right)-n_{t}\left(x\right)\right]$$

Ψ indicates the electrostatic potential, ε indicates the dielectric constant, q is the charge of an electron, n is the concentration of electrons, p is the concentration of holes, ${N_{A}}^{-}$ represents ionized acceptor density, ${N_{D}}^{+}$ signifies ionized donor density, $p_{t}$ and $n_{t}$ represent hole and electron traps, respectively, and x is the position coordinate.^49^

Hole (Equation 4) and electron (Equation 5) continuity equations:

(Equation 4) $$\frac{dp_{n}}{dt}=G_{p}-\frac{p_{n}-p_{n0}}{\tau_{p}}+p_{n}\mu_{p}\frac{dE}{dx}+\mu_{p}E\frac{dP_{n}}{dx}+D_{p}\frac{d^{2}p_{n}}{dx^{2}}$$

(Equation 5) $$\frac{dp_{p}}{dt}=G_{n}-\frac{n_{p}-n_{p0}}{\tau_{n}}+n_{p}\mu_{n}\frac{dE}{dx}+\mu_{n}E\frac{dn_{p}}{dx}+D_{n}\frac{d^{2}n_{p}}{dx^{2}}$$

$G_{n}$ and $G_{p}$ indicate generation rates of electrons and holes, $n_{p}$ and $p_{n}$ express the concentrations of electrons and holes in the p and n region, respectively, $n_{p0}$ and $p_{n0}$ indicate equilibrium concentrations of holes and electrons in the p and n region, respectively, $\tau_{n}$ and $\tau_{p}$ state a lifetime of electrons and holes, $\mu_{n}$ and $\mu_{p}$ indicate the mobility of electrons and holes, respectively, E signifies the electric field, and $D_{n}$ and $D_{p}$ denote the diffusion coefficient of electrons and holes. The carrier transport acquired because of drift and diffusion for electrons and holes is stated in Equations 6 and 7,^50^:

(Equation 6) $$J_{n}\;\left(x\right)=qn\mu_{n}E+qD_{n}\frac{dn}{dx}=n\mu_{n}\frac{dE_{Fn}}{dx}$$

(Equation 7) $$J_{p}\;\left(x\right)=qp\mu_{p}E-qD_{p}\frac{dp}{dx}=n\mu_{p}\frac{dE_{Fp}}{dx}$$

$E_{Fn}$ and $E_{Fp}$ represent the quasi-Fermi levels of the electron and hole, respectively.

### Effect of the conduction band offset (CBO)

The difference between the electron affinities of semiconductors determines the conduction band offset ($CBO=\Delta E_{C}$) and valence band offset (VBO) that are expressed by Equation 8 and Equation 9:

(Equation 8) $$CBO=\Delta E_{C}=\chi_{absorber}-\chi_{buffer}$$

(Equation 9) $$VBO=\left(\chi_{absorber}+{Eg}_{absorber}\right)-\left(\chi_{buffer}+{Eg}_{buffer}\right)$$

The type of barrier at the interface between the absorber and buffer layer is of great importance to the performance of solar cells. If CBO is positive ($\chi_{abs}>\chi_{buff}$), a spike-like barrier is formed, and the electrons undergo recombination at the interface^51^ and are prevented from passing to the other side. Thus, the photo-generated electrons must utilize kinetic energy to overcome the barrier at the hetero-junction interface. CBO is negative ($\chi_{abs}<\chi_{buff}$), a cliff like barrier occurs, and a small energy separation occurs between the valence and conduction bands at the interface.^52^ Still, photo-generated electrons gain kinetic energy to overcome the barrier.^53^ However, narrowing the band gap at the interface can lead to the recombination of charge carriers at the interface.^49^

**Figure 6:**
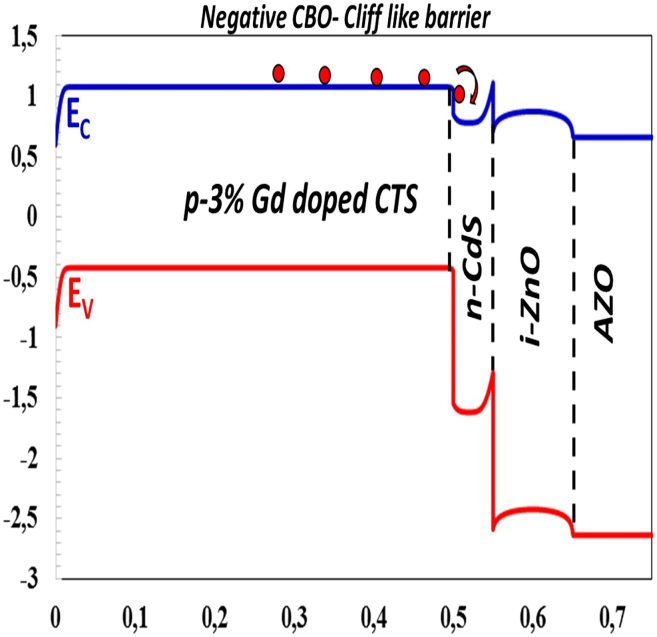
Negative CBO leads to cliff like barrier

**Figure 7:**
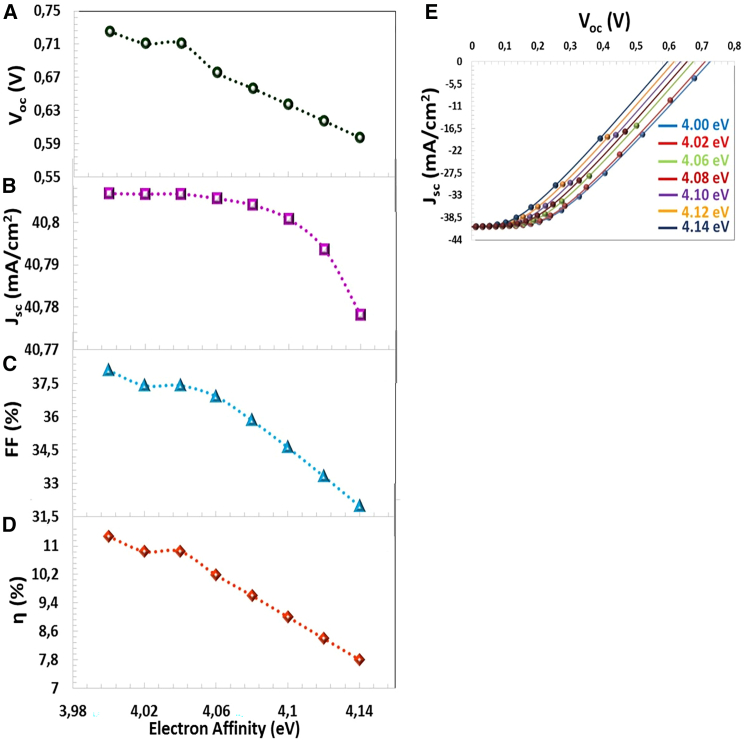
Electron Affinity Effect (A) $V_{OC}$, (B) $J_{SC}$, (C) FF, (D) η photovoltaic parameters, and (E) J−V characteristics depending on the electron affinity (χ).

In this study, we calculated the photovoltaic parameters for the electron affinity of CTS-4 between χ = 4.00 eV and χ = 4.14 eV ($\Delta E_{c}$ = (−0.2 eV) and (−0.06 eV)), as seen in Figure 6. For $\Delta E_{c}$ = −0.2 eV and −0.06 eV (for χ = 4.00 eV and χ = 4.14 eV), cliff-like CBO occurs formed close to the flat band in Figure 6. VBO (−1.1 eV and 0.96 eV) values are negative and small value that results to a cliff band gap which limits the recombination of the photo-excited charger carriers at the absorber-buffer interface.^52^ This shows that the semiconductors in the hetero-junction are in good alignment.^54^ As the electron affinity of the CTS-4 absorber layer decreases, the conduction band discontinuity decreases and forms a small barrier against photo-excited charge carriers.^55^ The easy transfer of charge carriers causes an increase in charge collection and improves efficiency. According to Figures 7A–7E, with decreasing electron affinity, all photovoltaic parameters increased, and an ideal J−V characteristic was obtained. χ = 4.00 eV was the optimal value and resulted in the highest PV parameters ($J_{sc}=40.8068\;mA/{cm}^{2}$, $V_{oc}=0.725\;mV$, FF=38.10%, η=11.28%).

### The influence of the interface defect density ($N_{t}$)

**Figure 8:**
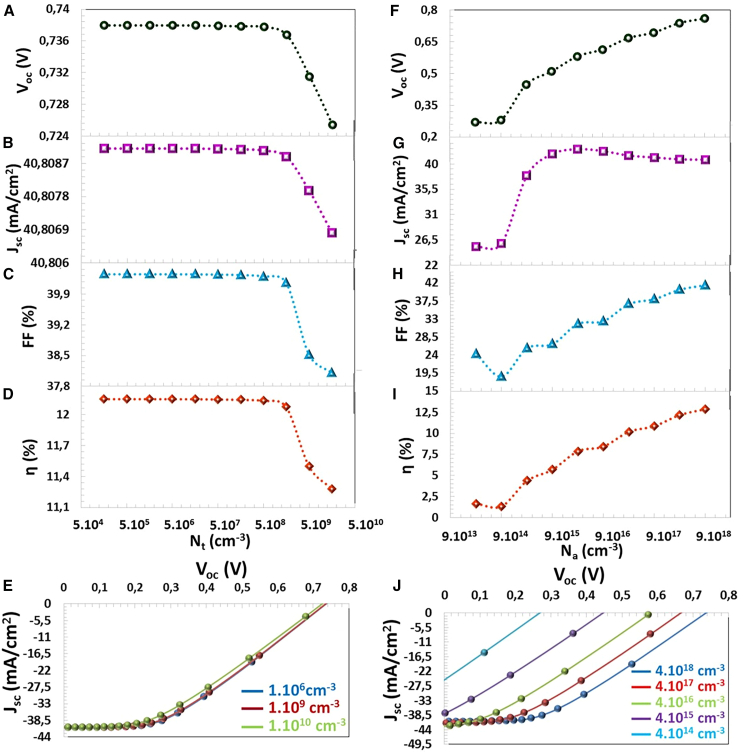
Defect-Level Influence (A) $V_{OC}$, (B) $J_{SC}$, (C) FF, (D) η photovoltaic parameters, (E) J−V characteristics depending on the $N_{t}$ and (F) $V_{OC}$, (G) $J_{SC}$, (H) FF, (I) η photovoltaic parameters, and (J) J−V characteristics depending on the shallow acceptor density ($N_{a}$).

Structural defects, pinholes, dangling bonds in the material during thin film production, lattice mismatch, electron affinity mismatch,^56^ and band incompatibility between the two semiconductors lead to interface defects. Interfacial defect density leads to traps that act as recombination centers. Higher defect density implies these defect regions capture more electrons.^55^ In addition, interface defects can cause increased series resistance and low efficiency.^57^ All photovoltaic parameters were changed for $N_{t}$ <5.10^8^ cm^−3^, but these parameters decreased for $N_{t}$ >5.10^8^ cm^−3^. According to Figures 8A–8D, when $N_{t}$ value risen from 1.10^5^ cm^−3^ to 1.10^10^ cm^−3^, $V_{OC}$, FF and η values, which dropped from 0.7379 V to 0.7253 V, from 40.35% to 38.10%, and from 12.15% to 11.28%, respectively. However, the rise in $N_{t}$ value did not cause a significant decrease in $J_{SC}$ value of the solar cell.

### The influence of the acceptor defect density in the active layer

Acceptor defects in the semiconductor lattice are factors that enhance *p*-type conductivity. There are the acceptor defects that copper vacancies ($V_{CU}$), Gd occupied $V_{CU}$ in CTS-4 semiconductor. In this study, photovoltaic parameters of the solar cell were calculated for between $N_{a}$ = 3.18×10^10^ and 8.18×10^14^ cm^−3^. With increasing acceptor density, the $V_{OC}$ value increment as the saturation current of the device increases and photocurrent decreases because of the dominance of the recombination of photo generated hole pairs.^32^^,^^55^ It is also expressed by the Equation 10 that $V_{OC}$ depends on the acceptor defect density (*p* doping density).

(Equation 10) $$V_{oc}=\frac{k_{B}T}{q}\ln\left(\frac{np}{n_{i}^{2}}\right)$$

T is the absolute temperature, q is the electron charge, $k_{B}$ is the Boltzmann constant, n(p) is the electron (hole) concentration under illumination, and $n_{i}$ is the intrinsic concentration of both electrons and holes.

**Figure 9:**
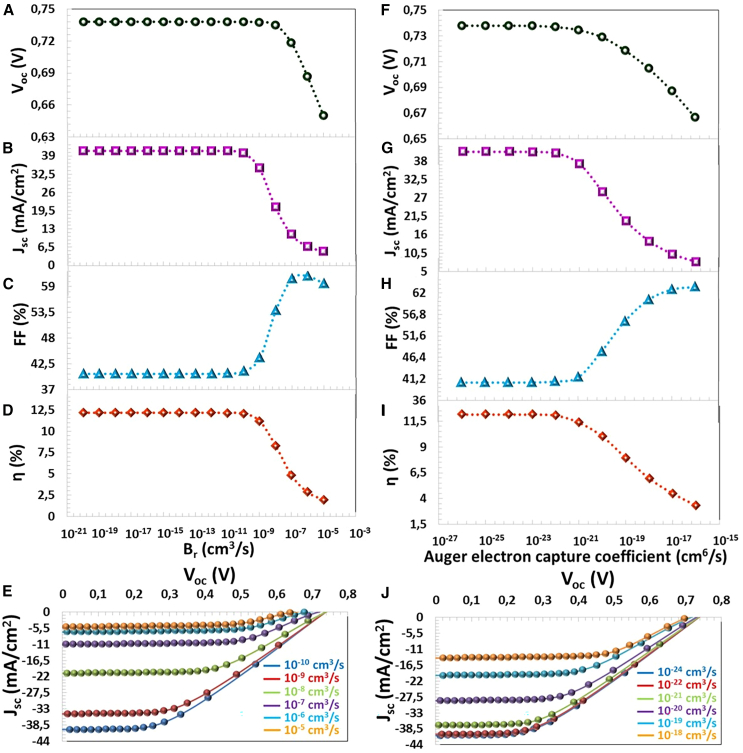
Recombination Dynamics (A) $V_{OC}$, (B) $J_{SC}$, (C) FF, (D) η photovoltaic parameters, (E) J−V characteristics depending on the radiative recombination coefficient ($B_{r}$), (F) $V_{OC}$, (G) $J_{SC}$, (H) FF, (I) η photovoltaic parameters, and (J) J−V characteristics depending on the Auger electron capture coefficient

The p doping concentration (the acceptor defect density) increases, and the hole number rises in the thin CTS film with Gd element doping. Increasing the $N_{a}$ value causes the charge carrier density to increase in the semiconductor and the depletion region charge accumulation development. So, when $N_{a}$ value risen from 4.11×10^14^ cm^−3^ to 9.12×10^18^ cm^−3^, $V_{OC}$, $J_{SC}$, FF and η values, which increased from 0.269 V to 0.759 V, from 25.31 mA/cm^2^ to 40.75 mA/cm^2^, from 24.26% to 41.46%, and from 1.65% to 12.83%, respectively. For the $N_{a}$ = 4.12×10^18^ cm^−3^ of the experimentally produced CTS-4 thin film, $V_{OC}$ = 0.737 V, $J_{SC}$ = 40.80 mA/cm^2^, FF = 40.35% η = 12.15%, as represented in Figures 9F–I.

### Effect of the radiative recombination

A photon is emitted as the electron in the conduction band moves to the valence band. In this process, the recombination of electrons and holes is called radiative recombination.^58^^,^^59^ The radiative recombination ($B_{r})$ is expressed by Equation 11.

(Equation 11) $$B_{r}=\frac{1}{\tau_{n\;or\;p,rad}N_{A\;or\;D}}$$

$B_{r}$ ($\frac{{cm}^{3}}{s}$) is the radiative recombination coefficient, $\tau_{n,rad}\left(s\right)$ and $\tau_{p,rad}\left(s\right)$ which are the radiative lifetime of the electron and the hole carriers, respectively.^58^ In this study, the efficiency of CTS-4 solar cells did not change between $B_{r}$ = 10^−17^ cm^3^/s and 10^−10^ cm^3^/s, when $B_{r}$ increased from 10^−10^ cm^3^/s to 10^−5^ cm^3^/s, $V_{OC}$, $J_{SC}$, FF and η photovoltaic parameters of the solar cell decreased from 0.737 to 0.650 V, from 40.80 mA/cm^2^ to 5.11 mA/cm^2^, and from 12.15% to 1.98%, as seen in Figures 9A–9E. According to Equation 11, the increase in $B_{r}$ that causes the lifetime or carrier density of the charge carriers to decrease, and this reduces the performance of the solar cell.

**Figure 10:**
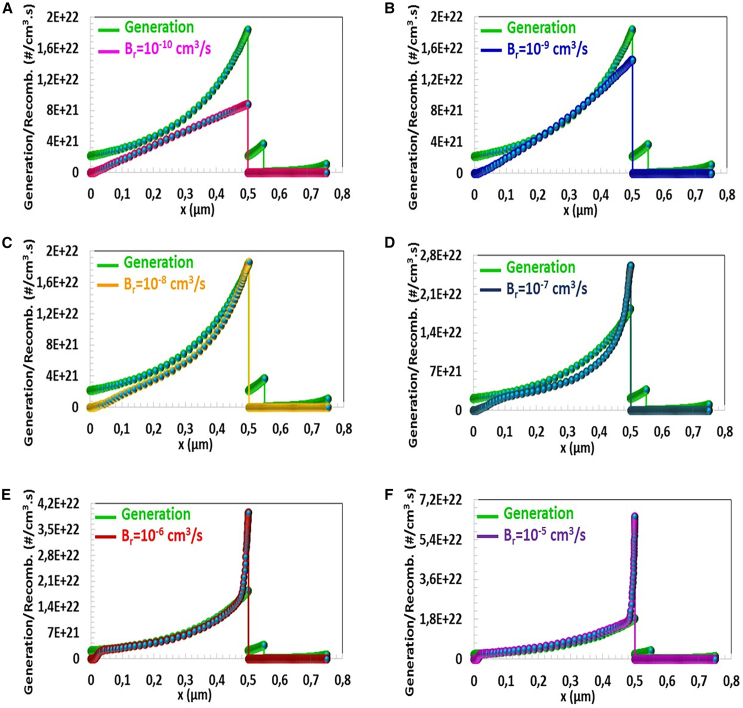
Generation/Recombination-Position (x) characteristic of CTS-4 solar cell for the radiative recombination coefficients ($B_{r}$) between10^−10^ cm^3^/s-10^−5^ cm^3^/s

In solar cells, the high density of photo-excited charge carriers at 1.83×10^22^ (#/cm^3^.s) is formed in the region close to the depletion region of the *p*-type absorber semiconductor (x = 500 μm), as seen in Figure 10. Since light absorption decreases from the depletion region to the back contact region, charge generation decreases to 2.12×10^21^ (#/cm^3^.s) (x=0 μm). When Br was increased from 10^−10^ cm^3^/s to 10^−9^ cm^3^/s, radiative recombination at the side of the depletion region increased from 8.77×10^21^ (#/cm^3^.s) to 1.45×10^22^ (#/cm^3^.s). Since this recombination rate remained below the charge formation, J−V characteristic did not change significantly, as seen in Figure 10E). After $B_{r}$ = 10^−9^ cm^3^/s (between 10^−8^ and 10^−5^ cm^3^/s), photovoltaic performance deteriorated significantly as the recombination rate exceeded the charge generation, as seen in Figures 10C–10F.

### Effect of the Auger electron recombination

The electron and hole recombine in the absorber semiconductor and release energy. This energy is transferred to electrons and holes without radiation, allowing them to adapt to higher energy states. This event is called Auger recombination (non-radiative process). Auger recombination coefficient is stated using Equation 12^59^:

(12) $$B_{Auger,n\;or\;p}=\frac{1}{\tau_{n\;or\;p,rad}N_{A\;or\;D}^{2}}$$

**Figure 11:**
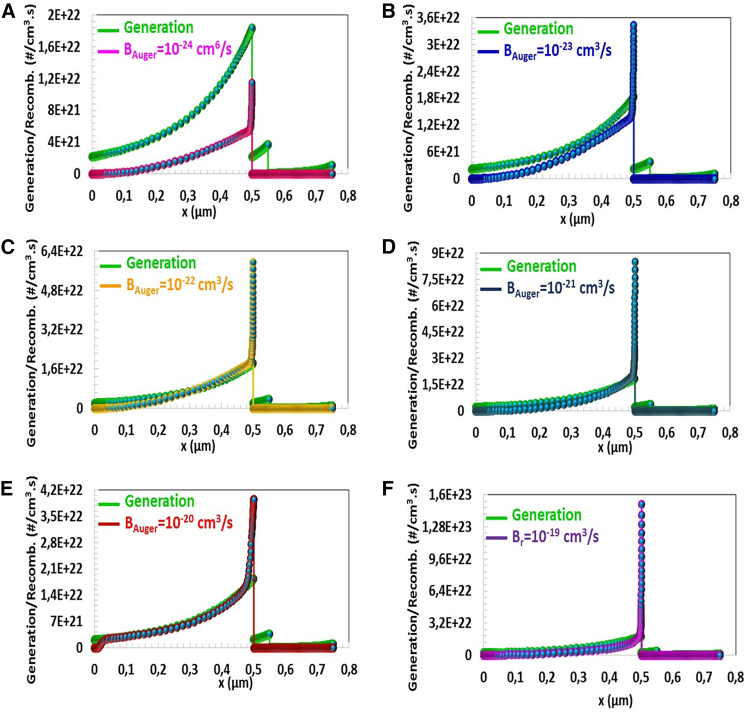
Generation/Recombination-Position (x) characteristic of CTS-4 solar cell for the Auger electron recombination coefficients ($B_{\text{Auger}}$) between10^−24^ cm^6^/s-10^−19^ cm^6^/s

This study calculated photovoltaic parameters depending on the Auger recombination coefficient between 10^−26^ cm^6^/s and 10^−16^ cm^6^/s. All parameters showed a significant change up to 10^−23^ cm^6^/s. When the Auger recombination coefficient increased from 10^−23^ cm^6^/s to 10^−16^ cm^6^/s in Figure 11, $V_{OC}$, $J_{SC}$, and η photovoltaic parameters of the solar cell decreased from 0.737 to 0.666 V, from 40.80 mA/cm^2^ to 7.83 mA/cm^2^, and from 12.15% to 3.31%, respectively. According to Figure 11, while the J−V characteristics are close to each other at 10^−24^ cm^6^/s and 10^−22^ cm^6^/s, there is a significant deterioration in the J−V curves after $B_{Auger,n}$ 10^−20^ cm^6^/s.

According to the characteristic in Figure 11, for $B_{\text{Auger}}{=10}^{-24\;}$ cm^6^/s, the amount of the recombination of the charges is relatively low compared to the charge generation. For $B_{\text{Auger}}{=10}^{-22}$ cm^6^/s and above, the photovoltaic performance and, therefore, J−V curve deteriorated as the amount of the recombination of the charges exceeded that of generation.

### Effect of the defect density in the absorber layer

The strain of the absorber’s thin film, the formation of pinholes and cracks, and the number of grain boundaries lead to the formation of defects and traps and reduce the lifetime of the carriers. Thus, the stability of the thin film decreases, and the degradation rate increases. As the degradation rate increases, the defect density rises, which may cause Shockley-Read-Hall recombination (SRH) to dominate in the absorber layer.^60^ The SRH model can express the effect of defect density. Electrons pass between bands through new energy states (localized states) formed in the band gap by defects. These energy states are referred to as SRH trap-assisted recombination.^61^

(Equation 13) $$R=\frac{np-n_{i}^{2}}{\tau_{p}\left(n+N_{C}e^{\frac{\left(E_{g}-E_{t}\right)}{k_{B}T}}\right)+\tau_{n}\left(p+N_{V}e^{\frac{\left(E_{t}\right)}{k_{B}T}}\right)}$$

*p* and n are the concentrations of the mobile holes and electrons. $E_{t}$ refers to the energy level where trap defects are located. Defect density is denoted in Equation 14 gives information about the carrier recombination rate (R).^62^ $\tau_{n,p}$ express the lifetime of the electrons and holes, which can be obtained using Equation 13^63^:

(Equation 14) $$\tau_{n,p}=\frac{1}{\sigma_{n,p}.\nu_{th}.N_{t}}$$

$\sigma_{n,p}$ is capturing the cross-section area of the electrons and holes. $N_{t}$ is the density of the trap defect and $\nu_{th}$ is the thermal velocity. The relation between the lifetime and the diffusion length is defined by Equation 15^63^:

(Equation 15) $$L_{n,p}=\sqrt{\frac{\mu_{n,p}k_{B}T}{q}\tau_{n,p}}$$

**Figure 12:**
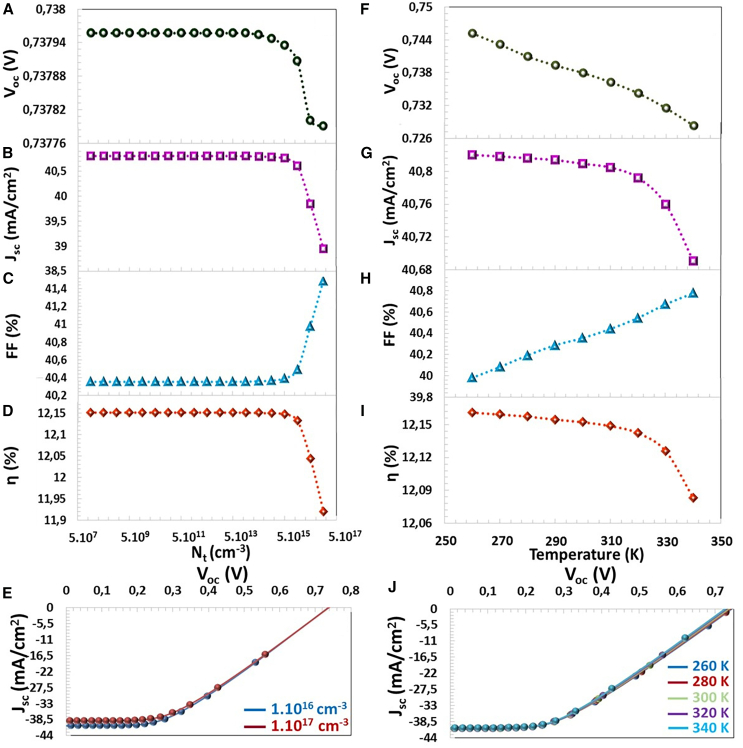
Temperature-Defect Interplay (A) $V_{OC}$, (B) $J_{SC}$, (C) FF, (D) η photovoltaic parameters, (E) J−V characteristics depending on $N_{t}$ (cm^−3^) in the CTS-4 absorber layer and (F) $V_{OC}$, (G) $J_{SC}$, (H) FF, (I) η photovoltaic parameters, and (J) J−V characteristics depending on the operating temperature.

$\mu_{n,p}$ is the electron and hole mobility, and q is the carrier charge. The equations show that the increase in the defect density in the semiconductor causes the recombination of the carrier charges, reducing the carrier lifetime and decreasing the performance of solar cells.^62^^,^^64^ According to Figures 12A–12D, there was no significant change in the photovoltaic parameters from $N_{t}$ = 1.10^8^ to 3.10^13^ cm^−3^ value. However, after 3.10^13^ cm^−3^ value, although FF values increased, and a decrease was observed in another photovoltaic parameter. When $N_{t}$ was risen from 5.10^13^ cm^−3^ to 1.10^17^ cm^−3^, $V_{OC}$, $J_{SC}$, and η parameters, which decreased from 0.7379 V to 0.7377 V, from 40.80 mA/cm^2^ to 38.94 mA/cm^2^, and from 12.15% to 11.91%, respectively, as given in Figure 12E. The defects present in the absorber semiconductor act as recombination points for electron and hole pairs. As the density of defects increases, minority charge carriers undergo recombination, thus reducing their diffusion length and lifetime. This leads to a decrease in $V_{OC}$, $J_{SC}$, and η values.

**Figure 13:**
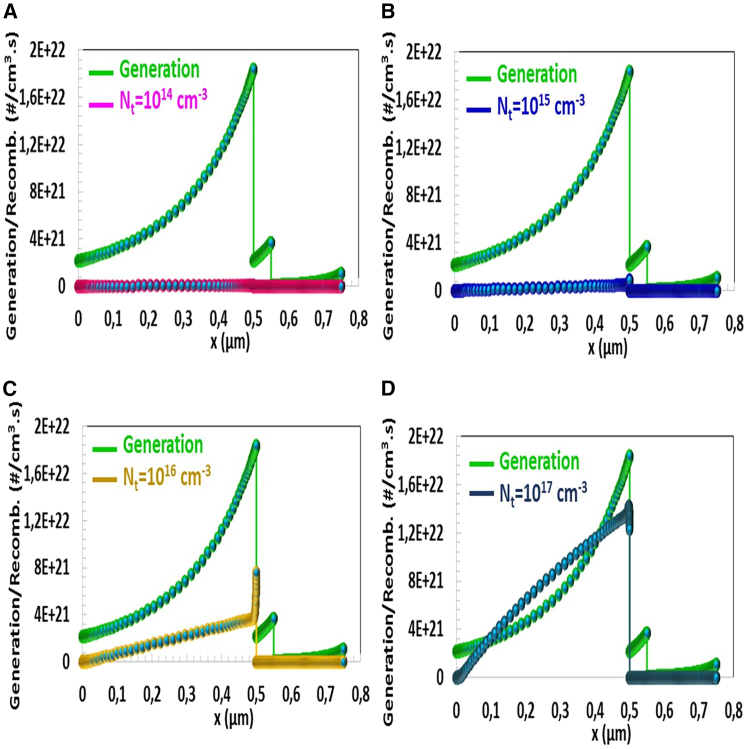
Generation/Recombination-Position (x) characteristic of CTS-4 solar cell for the defect density in the absorber layer between 10^8^ cm^−3^ and 10^17^ cm^−3^

According to the generation/recombination-position graph of the active layer cell for the defect density ($N_{t}$) in the absorber layer between 10^14^ cm^3^ and 10^17^ cm^3^, shown in Figure 12, the amount of SRH recombination is lower than the amount of the generation up to $N_{t}$ = 10^16^ cm^−3^, and the J−V curve does not show an evident change in Figure 12E). However, for $N_{t}$ = 10^17^ cm^−3^, the SRH recombination rate was high and negatively affected the photovoltaic performance between x = 0.1 μm and 0.4 μm range. $N_{t}$ increases, the diffusion length ($L_{n})$ of the minority charges carrier decreases and their lifetime ($\tau_{n}$) decreases, as seen in Figure 13 and Table 3. In particular, when N increased from 10^13^ cm^−3^ to 10^17^ cm^−3^, $L_{n}$ and $\tau_{n}$ decreased from 5.1 × 10^1^ μm to 5.1 × 10^−1^ μm and from 10^4^ ns to 10° ns, respectively. This reduced the charge accumulation in the solar cell and all photovoltaic parameters.

**Table 3:** Electron/Hole diffusion Length, Electron/Hole lifetime depending on the defect density in the absorber layer

$N_{t}$ (cm^−3^)	Electron diffusion Length ($L_{n}$) (μm)	Hole diffusion Length ($L_{p}$) (μm)	Electron lifetime ($\tau_{n})\left(ns\right)$	Hole lifetime ($\tau_{p})\left(ns\right)$
10^13^	5.1×10^1^	3.9×10^1^	10^4^	10^4^
10^14^	1.6×10^1^	1.2×10^1^	10^3^	10^3^
10^15^	5.1×10^0^	3.9×10^0^	10^2^	10^2^
10^16^	1.6×10^0^	1.2×10^0^	10^1^	10^1^
10^17^	5.1 × 10^−1^	3.9 × 10^−1^	10^0^	10^0^

### Operating temperature effects

The operating temperature has a significant impact on the performance of solar cells. As seen in the Figures 12F–12I, when the temperature was increased from 200 K to 400 K, $V_{OC}$, $J_{SC}$, and efficiency decreased, but FF increased. With the increasing temperature, the intrinsic carrier concentration is higher, and the semiconductor band gap is reduced. Thus, to the reverse saturation current density augmentation, there is a decrease in $V_{OC}$. The narrowing of the band gap (negative $dE_{g}/dt)$ with increasing temperature accelerates the recombination of electron-hole pairs between the conduction and valence bands of the semiconductor a, increasing the dark current in the solar cell.^65^ The relationship between the energy band gap of the semiconductor, the operating temperature, and $V_{OC}$ value is expressed by the following equation.^64^

(Equation 16) $$\frac{d\left(V_{oc}\right)}{dT}=\frac{\left(V_{oc}-\frac{E_{g}}{q}\right)}{T}$$

The increase in temperature imparts thermal energy to the electrons, and the electrons become thermally excited. Thus, electrons move quickly to the conduction band, causing an indirect reduction in the band gap. According to Equation 16, as the temperature increases, the band gap and $V_{OC}$ value decrease.

In addition, as the thermal energy increases, the interatomic vibration frequency increases and grows in the interatomic space. Increasing the interatomic space causes the potential energy of the electron to decrease and, therefore, the energy band gap to decrease.^66^ Increasing temperature results in stress and strain formation, enhancing the interface defects.^67^ Hence, the interlayer connections weaken. Defect formations at the interface lead to recombination formations and decreased diffusion length. This results in an almost linear decrease in $V_{OC}$ and efficiency.^68^

### Series and the shunt resistance effects

The most common parasitic resistances of solar cells are the series ($R_{s}$) and the shunt resistance ($R_{sh}$). $R_{s}$ refers to the resistance between the metal contact and the semiconductor, the resistance resulting from current movements between the two layers, and other internal resistances. Furthermore, the surface strain, grain boundaries, interface traps, and thin film surface resistance lead to $R_{s}$. Defects that occur during production, pinholes or crack formations, losses due to alternating current paths, and leakage current in the joint area cause $R_{sh}$ resistance. The effect of $R_{s}$ and $R_{sh}$ resistances on photovoltaic parameters can be expressed with the Equation 17:

(Equation 17) $$I=I_{ph}-I_{o}\left[\exp\left(\frac{q\left(V+IR_{s}\right)}{nkT}\right)-1\right]-\frac{V+IR_{s}}{R_{sh}}$$

I is out current, $I_{ph}$ is the light-generated current, $I_{o}$ is the saturation current, q is the elementary charge, V is the voltage, n is the ideality factor, k is the Boltzmann constant, and T is the temperature.

**Figure 14:**
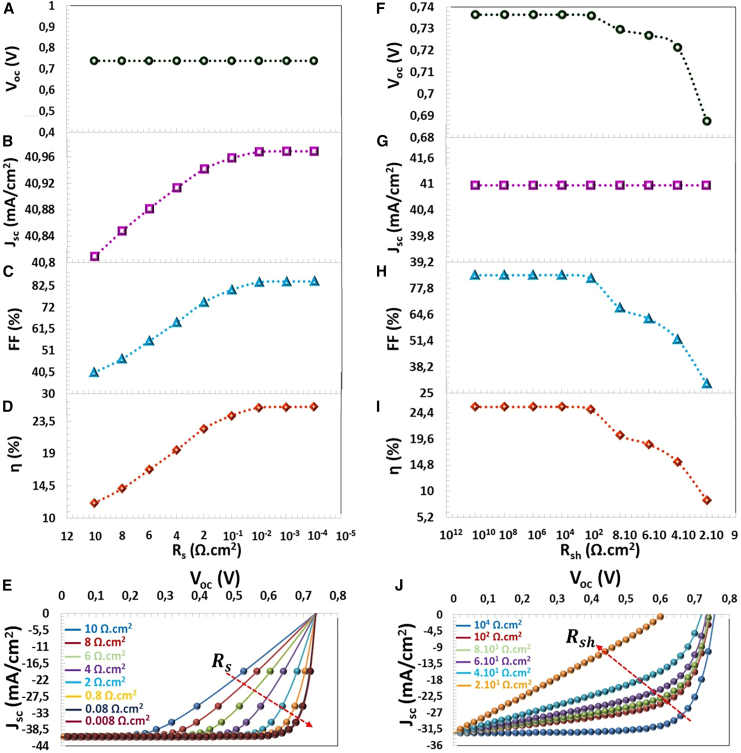
Resistance Effects (A) $V_{OC}$, (B) $J_{SC}$, (C) FF, (D) η photovoltaic parameters, (E) J−V characteristics depending on the Series resistance (Ω.cm^2^), (F) $V_{OC}$, (G) $J_{SC}$, (H) FF, (I) η photovoltaic parameters, and (J) J−V characteristics depending on Shunt resistance (Ω.cm^2^).

In this theoretical study, $R_{s}$ was changed from 8 Ω cm^2^ to 8 × 10^−4^ Ω cm^2^, the value of $R_{sh}$ was changed from 4 × 10^1^ Ω cm^2^ to 1 × 10^8^ Ω cm^2^. While the decrease in $R_{s}$ resistance did not cause a significant change in the $V_{OC}$, parameter, but $J_{SC}$, FF and efficiency parameters increased, as seen in Figures 14A–14D. High series resistance may have reduced the $J_{SC}$ value because it prevented the movement of charge carriers.^68^ In addition, it should be noted that the photovoltaic parameters are fixed between 8 × 10^−2^ Ω cm^2^ and 8 × 10^−4^ Ω cm^2^ series resistance values. When $R_{s}$ was reduced from 10^1^ Ω cm^2^ to 8 × 10^−4^ Ω cm^2^, $J_{SC}$, FF and η parameters, which augmented from 40.80 mA/cm^2^ to 40.96 mA/cm^2^, from 40.35% to 84.46% and 12.15%–25.28%, respectively. With the increase in shunt resistance, the current passing through the shunt (or any parallel shunt) decreases, and this causes an increase in $J_{SC}$ and $V_{OC}$, values. The higher $R_{sh}$ that increases $V_{OC}$, and FF values as seen in Figures 14F–14I. For infinite $R_{sh}$, the output voltage can be expressed by the following equation:

(Equation 19) $$V=\frac{kT}{q}\ln\left(\frac{I_{sc}}{I_{o}}+1\right)$$

Reverse saturation current negatively affects the output voltage. Equation 19 explains this logic mathematically. $I_{0}$ There is evidence of a leakage current across the junction region due to faults occurring during thin film production.^67^ Therefore, all photovoltaic parameters of the solar cell were reduced with the decrease the $R_{sh}$ as seen in Figures 14F–14L. As a result, for $R_{s}$ = 8 × 10^−4^ Ω cm^2^ and $R_{sh}$ = 8 × 10^4^ Ω cm^2^, the solar cell exhibited the most improved photovoltaic performance.

### Back contact materials

**Figure 15:**
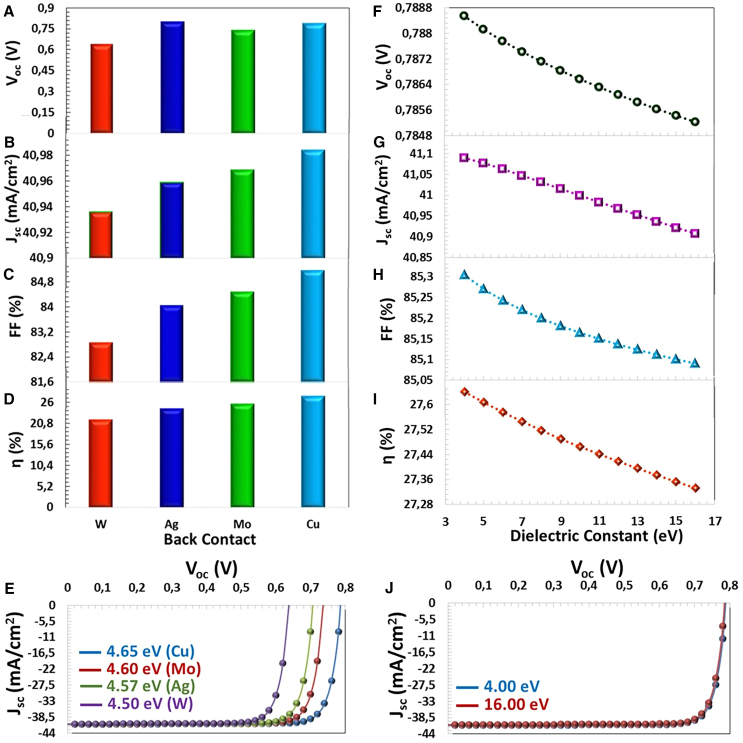
Contact and Dielectric Impact (A) $V_{OC}$, (B) $J_{SC}$, (C) FF, (D) η photovoltaic parameters, (E) J−V characteristics depending on the work function of the back contact metal (eV), (F) $V_{OC}$, (G) $J_{SC}$, (H) FF, (I) η photovoltaic parameters, and (J) J−V characteristics depending on dielectric permittivity (eV).

The Figures 15A–15E shows the calculated efficiency values and J−V characteristics depending on the back contact materials with work functions between 4.50 eV and 5.00 eV. It has been observed that there is a significant increase in the efficiency of thin film solar cells with the rise of the work function, as seen in Table 4. If $\Phi_{M}>\Phi_{S}$ for *p*-type semiconductors, the back contact exhibits ohmic behavior. Among W ($\Phi_{W}=4,50$ eV), Ag ($\Phi_{Ag}=4,57$ eV) Mo ($\Phi_{Mo}=4,60$ eV), Cu ($\Phi_{Cu}=4,65$ eV) contact materials, Cu back contact shows slightly more ohmic behavior for *p* type Gd doped CTS ($\Phi_{Gd-CTS}=4.65$ eV) semiconductor. For a lower metal work function, the Schottky barrier forms between the metal and semiconductor and blocks the transition to the hole.^56^ Due to band bending at the metal and semiconductor interface with the increase of the work function,^60^ the barrier height between the semiconductor and the contact material decreases, making the hole (primary charge) the transition from the semiconductor to the back contact easier.^69^^,^^70^ Therefore, an ideal charge transfer is acquired in the rear contact area, which improves photovoltaic performance.

**Table 4:** The photovoltaic parameters of CTS-4 solar cells depend on the different metal back contacts

Back Metal	W	Ag	Mo	Cu
Work function (eV)	4.50	4.57	4.60	4.65
$V_{oc}$ (V)	0.637	0.796	0.736	0.786
$J_{sc}$ (mA/cm^2^)	40.93	40.95	40.96	40.98
FF (%)	82.85	84.03	84.46	85.15
η (%)	21.62	24.31	25.48	27.44

### Dielectric constant effects

The dielectric permittivity ($\varepsilon_{r}$) of the active layer (absorber layer) that relates to the composition of the absorber layer and the environment temperature. According to Poisson, the higher annealing temperatures differential equation $\nabla^{2}\Phi =$ higher annealing temperatures, the majority of carrier charges in the active layer are consumed, forming a significant potential in the depletion region and thus limiting the recombination of the charges.^71^ Due to a stable built-in potential, the reduction in SRH recombination determines the improved performance of the solar cell. The large $\varepsilon_{r}$ causes a small decrease in the electrical field, which weakens the separation of charge carriers and causes them to recombine. In this case, with an increase in the dielectric permittivity, the efficiency of the thin film solar cell is decreased, which is proven in this study, as seen in Figures 15F–15I. According to the calculations between $\varepsilon_{r}$ = 4 eV and 16 eV, the solar cell with photovoltaic parameters of $V_{oc}$ = 0.7885 eV, $J_{sc}$ = 41.09 mA/cm^2^, FF = 85.30% and η = 27.3% exhibited the highest performance for $\varepsilon_{r}$ = 4 eV. However, as can be seen in Figure 15L, there is no significant change in photovoltaic parameters with the increase in $\varepsilon_{r}$ parameter. In this study, the effect of Gd element on the morphology, crystal, optical and electrical properties of CTS material was investigated. As a result, the optimal Gd-doped CTS material was determined, and the solar cell based on this material was modeled. Depending on the parameters such as interface defect density, acceptor defect density, electron affinity, radiative recombination coefficient, Auger electron capture coefficient, series, and shunt resistance, the highest photovoltaic parameters were determined for the best conditions and the lower ones for the less favourable conditions. These results are consistent with many studies reported in the literature.^66^^,^^72^^,^^73^^,^^74^

### Conclusions

This research focused on synthesizing Gd-doped CTS thin films using the spin coating. It evaluated the comprehensive impact of the Gd/Sn doping ratio on their crystallographic, optical, structural, and electrical properties, as well as photovoltaic performance. Initially, the predominant cubic phase (112) identified in the CTS-1 film underwent a significant transformation with an increased Gd/Sn doping ratio, leading to the disappearance of the (112) peak and the emergence of dominant CuS peaks. The value of crystallite size is found to be 53.37 and 51.59 nm for (112) orientation for CTS-1 and CTS-3 thin film, respectively, and 43.30, 50.30, and 45.87 nm for (106) orientation for CTS-1, CTS-2, and CTS-4 thin film, respectively due to an increase in crystallite defects and lattice mismatching between the host and dopant ions. The morphology of the CTS films varied from spherical grains to polygonal structures. Increased Gd concentration in the solution notably induced the formation of larger clusters and a substantial alteration in the surface topology of the films. Cu/Sn atomic ratio is changed between 2.19 and 3.80. The difference in the thin films' Cu/Sn atomic ratio may be attributed to Sn-loss resulting from the increasing Gd/Sn doping ratio. The energy band gap values observed: 2.00, 2.05, 2.10, and 1.50 eV, suggest the presence of SnS_2_ and CuS secondary phases. In terms of electrical properties, the initial free hole concentration of approximately 10^20^ cm^−3^ in the CTS-1 sample decreased to around 10^18^ cm^−3^ upon Gd doping, attributed to the high melting point of the Gd-doped CTS solution reducing the hole concentration in pure Cu_2_SnS_3_ films. The resistivity of the obtained films changed between 18.15x10^−3^ Ω-cm and 50.60x10^−2^ Ω-cm. We also modeled a Cu/*p*-Gd doped CTS/*n*-CdS/i-ZnO/ITO thin film solar cell configuration using the SCAPS-1D software, observing a cliff-like conduction band offset at the interface. The CTS-4 thin film solar cell showcased superior photovoltaic performance, characterized by parameters including an interface defect density $N_{t}$ <5.10^8^ cm^−^, acceptor defect density $N_{a}$ = 4.12×10^18^ cm^−3^ in the absorber layer, radiative recombination coefficient Br < 10^−9^ cm^3^/s, Auger recombination coefficient B_Auger_ < 10^−20^ cm^6^/s, defect density in the absorber layer $N_{t}$ <3.10^13^ cm^−3^, series resistance $R_{s}$ = 8 × 10^−4^ Ω cm^2^, shunt resistance $R_{sh}$ = 8 × 10^4^ Ω cm^2^, a work function of the back contact (copper) at 4.65 eV, and a dielectric constant $\varepsilon_{r}$ = 4 eV. Firstly, for χ = 4.00 eV electron affinity, PV parameters are $J_{sc}=40.8068\;mA/{cm}^{2}$, $V_{oc}=0.725\;mV$, FF=38.10%, η=11.28%. Then, these optimized conditions yielded exceptional photovoltaic parameters, achieving an open-circuit voltage of 0.7885 eV, short-circuit current density of 41.09 mA/cm^2^, fill factor of 85.30%, and an efficiency (η) of 27.3%. This detailed investigation elucidates the transformative effects of Gd doping on CTS thin films and paves the way for enhancing photovoltaic technologies through optimal material and interface engineering. As a result of this study, a design guideline on how to utilize a CdS layer in conventional CTS solar cells is provided that will enhance performance and reduce absorption material costs significantly. Based on current findings, it is suggested that it may be possible to fabricate a low-cost thin-film solar cell based on CTS shortly.

### Limitations of the study

This study, while comprehensive, has certain limitations that should be considered. The spin coating technique, although low-cost and simple, may not ensure uniformity and reproducibility across large surface areas, which could limit scalability for industrial applications. The investigation was confined to a specific range of Gd/Sn doping ratios, and the results may not fully represent the effects of broader compositional variations. Furthermore, the electrical and photovoltaic characterizations were based on simulation data without experimental validation under operational conditions. Aspects such as interfacial quality, thermal stability, and environmental durability were not addressed in this work. These factors should be further explored to strengthen the practical relevance of the findings.

## Resource availability

### Lead contact

Further information and requests for resources and reagents should be directed to and will be fulfilled by the lead contact, Dr. Mohamed A. Basyooni-M. Kabatas (m.kabatas@tudelft.nl & m.a.basyooni@gmail.com).

### Materials availability

This study did not generate new unique reagents.

### Data and code availability

• This article does not report original code.
• Any additional information required to reanalyze the data reported in this article is available from the lead contact upon request.

## STAR★Methods

### Key resources table

**Table:** 

REAGENT or RESOURCE	SOURCE	IDENTIFIER
**Other**		

soda-lime glasses	Sigma-Aldrich	Cat# 3010001
copper(II) acetate	Sigma-Aldrich	Cat# 308173 or CAS: 6046-93-1
gadolinium(III) chloride	Sigma-Aldrich	Cat# 449598 or CAS: 13450-84-5
Tin(II)choloride	Sigma-Aldrich	CAS# 7772-99-8
thiourea	Sigma-Aldrich	Cat# T8656 or CAS: 62-56-6
absolute ethanol	Merck	Cat# 32221 or CAS: 64-17-5
acetic acid (glacial)	Merck	Cat# 100063 or CAS: 64-19-7

### Experimental model and study participant details

This study does not include animals, human participants, plants, microbe strains, cell lines, primary cell cultures.

### Method details

This study fabricated Cu_2_Sn_1-x_Gd_x_S_3_ (where x = 0, 0.25, 0.50, and 0.75) on soda-lime glasses (SLGs) using the spin coating deposition method. The precursor solution comprised 0.719 g of copper(II) acetate (Cu(CH_3_COO)_2_·H_2_O), 0.450 g of gadolinium(III) chloride (GdCl_3_·6H_2_O), 0.542 g of tin(II) chloride dehydrate (SnCl_2_·2H_2_O), and 0.228 g of thiourea (CH_4_N_2_S), prepared under optimized conditions. Each chemical was separately dissolved in a mixture of absolute ethanol (≥ 99.5%) and acetic acid (glacial) and stirred magnetically for 6 hours at room temperature on a magnetic stirrer. During the preparation of solutions, thiourea solution was poured into copper(II) acetate solution, then the corresponding amount of gadolinium(III) chloride solution was added to thiourea/copper(II) acetate solution, and finally, tin(II) chloride dehydrate was added. 0.1 ml triethanolamine was dropped into the mixed solution to adjust the pH value. This mixture was then stirred for an additional 60 min at room temperature until a homogeneous, clear solution was achieved.

Before deposition, the SLGs were cleaned thoroughly by boiling in a mixture of H_2_O, NH_3_, and H_2_O_2_ at 105°C, followed by a similar treatment with H_2_O, H_2_O_2_, and HCl, to remove any remaining impurities. The SLGs were then rinsed in deionized water for 10 minutes and air-dried. The final solution was fabricated onto the SLGs via spin coating at 1500 rpm for 70 seconds. Each layer was preheated to 220°C for 10 minutes before adding the next layer. This deposition process was repeated twelve times. Finally, the samples were annealed using 50 mg sulfur in an N_2_ atmosphere at 500°C in a quartz furnace. The obtained sample names are CTS-1, CTS-2, CTS-3, and CTS-4 for Cu_2_SnS_3_, Cu_2_Gd_0.25_Sn_0.75_S_3_, Cu_2_Gd_0.5_Sn_0.5_S_3_, and Cu_2_Gd_0.75_Sn_0.25_S_3_, respectively.

This study investigated the effect of various Gd doping concentrations on CTS crystallite and their morphological, optical, and electrical properties. Panalytical/Empyrean X-ray diffraction was used to characterize the crystalline properties of CTS samples that were systematically annealed at 500°C sulfur atmosphere for 60 min. ZEISS Geminisem 500 FESEM and Energy Dispersive X-ray spectroscopy (EDX) attached to the FESEM were used to determine the surface morphology of CTS samples. A Shimadzu UV-3600 Plus spectrophotometer examined the CTS thin film energy band gap and absorbance between 1100 and 300 nm. The electrical properties of the CTS samples were investigated at room temperature using an HMS 3000 Hall effect measurement system. To measure the electrical conductivity of the samples, a four-probe method was applied to an area of approximately 1.5 × 1.0 cm^2^. Contacts were made with silver paste on the surface of thin films, maintaining a point-to-point distance of about 0.5 cm.

### Quantification and statistical analysis

We used the SCAPS-1D software to calculate the solar cell parameters.

## Acknowledgments

The authors thank several key institutions and groups for their invaluable support throughout this research. We thank the Selçuk University Scientific Research Projects (BAP) Coordination Office for funding and support through projects numbered 21406007 and 22401108. We also acknowledge the contributions of Selçuk University’s High Technology Research and Application Center (İL-TEK) and the SULTAN Center for Infrastructures. Additionally, we are thankful for the support from Dicle University's Scientific Research Coordinators (DUBAP) under project number FEN.22.003. Special thanks to Dr. Marc Burgelman’s group at the University of Gent, Belgium, for allowing us to use the SCAPS-1D simulation program, which was crucial in our simulations. The collaboration and resources provided by these organizations have been essential to the success of our work.

## Author contributions

Şilan Baturay contributed to conceptualization, methodology, investigation, data curation, formal analysis, and writing the original draft. M. Zafer Koylu contributed to conceptualization, methodology, investigation, data curation, and validation. Mohamed A. Basyooni-M. Kabatas contributed to data curation, visualization, formal analysis, and writing, review and editing. Serap Yiğit Gezgin contributed to software, data curation, and visualization. H. Şükür Kılıç contributed to software, validation, writing, review and editing. All authors reviewed the final version of the article, accepted responsibility for its content, and approved its submission.

## Declaration of interests

The authors declare no competing interests.
